# Miscellaneous

**Published:** 1885-09

**Authors:** 


### MISCELLANEOUS.

M. WILTZ says that the proportion of ozone in the air of Paris last year was in inverse ratio to the mortality from cholera.

A WHITE cement made of a mixture of plaster of Paris and borax is called Parian. Four bushels of it, with as much clear sand, will cover ten superficial yards half an inch thick.

THE best method of recovering faded green damasks is to dye them with indigo, acid green and common or glauber salts, with sulghuric and picric acid, with moderate boiling.

IN New South Wales, a bridge 3,000 feet long, with piers to be sunk 170 feet in all below the tide, is to be built over the Hawkerbury river. It is to be a double track railway bridge and will cost $2,000,000.

AT Dijon, in the Botanical Garden, is a poplar tree 130 feet high, with a circumference near the earth of 46 feet, and at sixtee 1 feet above the earth of 21 feet. It is supposed, with good reason that it is 500 years old.

BARLEY was cultivated before any of the other cerials in Scandinavia, and the generic name corn was restricted to it. It was cultivated in Iceland from 870 to 1400, and has been found growing as far north as 65 degrees.

A PATENT is reported to have been taken out for making an imitation maple syrup. An extract is obtained by soaking hickory bark in water, and to this cane or glucose syrup is added. The product has the taste and smell of the genuine article.

